# The Identification of a Small Molecule Compound That Reduces HIV-1 Nef-Mediated Viral Infectivity Enhancement

**DOI:** 10.1371/journal.pone.0027696

**Published:** 2011-11-15

**Authors:** Nopporn Chutiwitoonchai, Masateru Hiyoshi, Philip Mwimanzi, Takamasa Ueno, Akio Adachi, Hirotaka Ode, Hironori Sato, Oliver T. Fackler, Seiji Okada, Shinya Suzu

**Affiliations:** 1 Center for AIDS Research, Kumamoto University, Kumamoto, Japan; 2 Department of Microbiology, Institute of Health Biosciences, The University of Tokushima Graduate School, Tokushima, Japan; 3 Pathogen Genomics Center, National Institute of Infectious Diseases, Tokyo, Japan; 4 Department of Infectious Diseases, Virology, University of Heidelberg, Heidelberg, Germany; Institut Pasteur, France

## Abstract

Nef is a multifunctional HIV-1 protein that accelerates progression to AIDS, and enhances the infectivity of progeny viruses through a mechanism that is not yet understood. Here, we show that the small molecule compound 2c reduces Nef-mediated viral infectivity enhancement. When added to viral producer cells, 2c did not affect the efficiency of viral production itself. However, the infectivity of the viruses produced in the presence of 2c was significantly lower than that of control viruses. Importantly, an inhibitory effect was observed with Nef^+^ wild-type viruses, but not with viruses produced in the absence of Nef or in the presence of proline-rich PxxP motif-disrupted Nef, both of which displayed significantly reduced intrinsic infectivity. Meanwhile, the overexpression of the SH3 domain of the tyrosine kinase Hck, which binds to a PxxP motif in Nef, also reduced viral infectivity. Importantly, 2c inhibited Hck SH3-Nef binding, which was more marked when Nef was pre-incubated with 2c prior to its incubation with Hck, indicating that both Hck SH3 and 2c directly bind to Nef and that their binding sites overlap. These results imply that both 2c and the Hck SH3 domain inhibit the interaction of Nef with an unidentified host protein and thereby reduce Nef-mediated infectivity enhancement. The first inhibitory compound 2c is therefore a valuable chemical probe for revealing the underlying molecular mechanism by which Nef enhances the infectivity of HIV-1.

## Introduction

Nef is a 25- to 30-kDa protein with no catalytic activity encoded by the HIV-1 genome [1]–[4]. Studies of HIV-1-infected patients have demonstrated Nef to be a critical determinant of the progression to AIDS: HIV-1 strains without an intact *nef* gene were frequently isolated from non-progressive long-term survivors [5], [6]. A subsequent study of HIV-1 transgenic mice confirmed the pathogenetic activity of Nef: targeted expression of the entire coding sequence of HIV-1 in CD4^+^ T cells and macrophages caused a severe AIDS-like disease in mice, which was completely abolished by disruption of the *nef* gene [7].

Nef is multifunctional. For instance, it accelerates the endocytosis of CD4 [8], [9], the primary entry receptor for HIV-1, which allows efficient viral release from host cells [1]–[4]. Nef also reduces the surface expression of MHC I through multiple mechanisms [10]–[13], which diminishes the recognition of infected cells by CTL [1]–[4]. Nef is also known to activate the Src kinase Hck [14]–[16], which causes an impaired macrophage response to the cytokine M-CSF [17], [18] or triggers cell fusion of HIV-1-infected macrophages [19]. Another hallmark function of Nef is the enhancement of the intrinsic infectivity of progeny viruses. This function of Nef is independent of CD4 downregulation and requires the presence of Nef in viral producer cells [20]–[23]. Moreover, this function appears to depend on an early step of the target cell infection process, as Nef is dispensable for the infectivity of HIV-1 pseudotyped with vesicular stomatitis virus glycoprotein VSV-G [24], [25]. However, Nef does not affect viral assembly or maturation, and it is still unclear how Nef enhances viral infectivity [26].

Thus far, only a few chemical compounds that interfere with the functions of Nef have been identified. Among them, a series of guanidine alkaloid analogs were found to be too toxic for cell-based assays [27]. A unique diphenylfuropyrimidine and its analogs were identified to be strong inhibitors of the Nef-dependent activation of Hck, but their primary target seemed to be Hck not Nef [28]. In contrast, the chemical compounds D1 and 2c directly target Nef. Betzi et al. identified D1 and showed that it reduced Nef-mediated MHC I, but not CD4, downregulation in a dose-dependent manner [29]. Subsequently, we identified 2c, the structure of which is distinct from that of D1, and showed that it almost completely inhibited the Nef-dependent activation of Hck [30] and significantly reduced Nef-mediated MHC I, but not CD4, downregulation [31]. The fact that 2c has the inhibitory effect on MHC I downregulation and Hck activation, but not on CD4 downregulation, agrees with the finding that MHC I downregulation and Hck activation are mediated by overlapping motifs or amino acids of Nef, which are distinct from those required for CD4 downregulation [3], [9], [14], [18]. However, none of these compounds have been tested for their ability to interfere with the enhancement of viral infectivity by Nef.

In contrast to its requirement for elevated *in vivo* viral load [5], [6], Nef is not essential for viral replication in *ex vivo* cell cultures. Nonetheless, Nef significantly enhances viral replication in primary CD4^+^ T cells and macrophages that have been exposed to HIV-1 prior to their stimulation with mitogens [32], [33], a function of Nef that is likely determined by enhancement of the initial infection with cell-free HIV-1 [34]. In this regard, a compound that can reduce viral infectivity would be a valuable chemical probe for revealing the underlying mechanism of this function of Nef. In this study, we identified 2c as the first small compound that has an inhibitory effect on Nef-mediated HIV-1 infectivity enhancement and reported its inhibitory mechanism.

## Results and Discussion

### 2c reduces the infectivity of wild-type HIV-1

**Figure 1:**
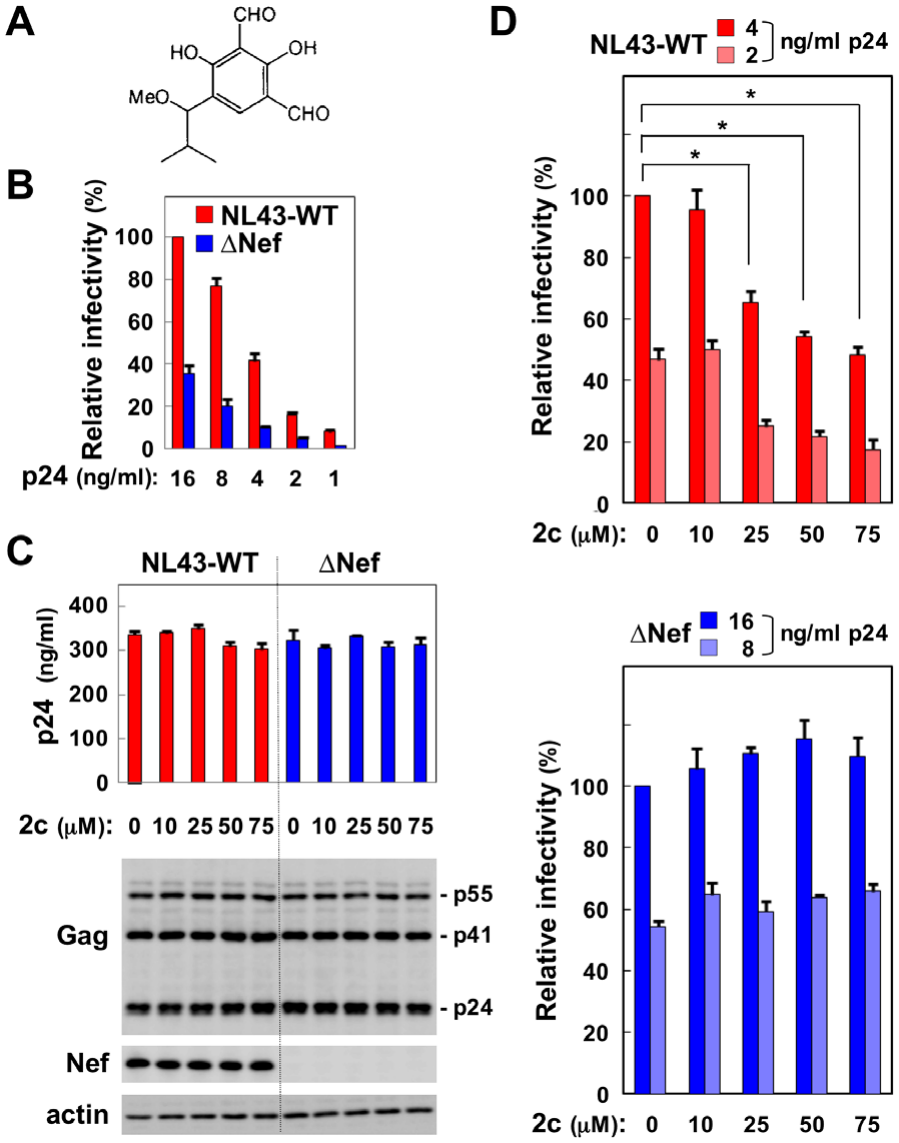
The effect of 2c on the infectivity of NL43 wild-type and Nef-defective mutant viruses. (A) The chemical structure of 2c. (B) The infectivity of the NL43 wild-type (WT) and Nef-defective mutant (ΔNef) viruses to the target TZM-bl cells was compared by varying the concentration of p24 Gag protein and is expressed as a percentage of the value for the sample on the far left. Data are shown as the mean±SD of triplicate assays and are representative of two independent experiments with similar results. (C) 2c was added to 293 cells producing NL43-WT or ΔNef viruses at the indicated concentrations for 2 days, and the concentration of p24 Gag protein in the cell supernatants was determined by ELISA (bar graph). Data are shown as the mean±SD of triplicate assays and are representative of two independent experiments with similar results. Alternatively, the producer cells were lysed and analyzed for the expression of Gag and Nef by Western blotting (lower blots). The actin blot was used as a loading control. (D) The infectivity of NL43-WT (upper) or ΔNef viruses (lower) produced by 293 cells in the absence or presence of the indicated concentrations of 2c was determined using TZM-bl cells as the target cells. The WT and ΔNef viruses were inoculated by changing the p24 concentration (2 or 4 ng/ml and 8 or 16 ng/ml for the WT and ΔNef viruses, respectively) so that the two viruses were similarly infective to the target cells. Infectivity is expressed as a percentage of the value for the sample on the far left. Data are shown as the mean±SD of triplicate assays and are representative of three independent experiments with similar results. *p<0.05.

**Figure 2:**
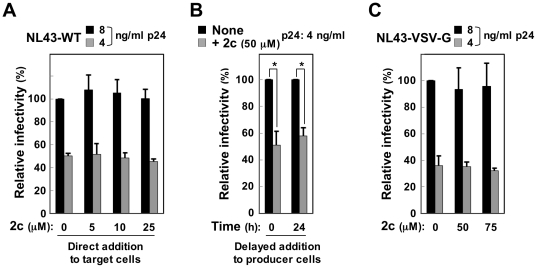
Several features of the activity of 2c on viral infectivity. (A) 2c was added to the target TZM-bl cells at the indicated concentrations together with the NL43 wild-type (WT) viruses produced in the absence of 2c. The amount of p24 inoculated was 4 or 8 ng/ml. The infectivity is expressed as a percentage of the value for the sample on the far left. (B) 2c (50 µM) or the control DMSO was added to the producer 293 cells immediately after transfection (0 h) or 24 h after transfection of the NL43 WT plasmid. The infectivity of the viruses was determined using TZM-bl cells and is expressed as a percentage of the value for the sample on the far left. The amount of p24 inoculated was 4 ng/ml. (C) 2c (50 or 75 µM) or the control DMSO was added to the producer 293 cells immediately after co-transfection of Env-defective NL43 plasmid and VSV-G expression plasmid. The infectivity of the pseudotyped viruses was determined using TZM-bl cells and is expressed as a percentage of the value for the sample on the far left. The amount of p24 inoculated was 4 or 8 ng/ml. (A–C) Data are shown as the mean±SD of triplicate assays and are representative of two independent experiments with similar results. *p<0.05.

We assessed the effect of the compound 2c (Fig. 1A) on Nef-mediated infectivity enhancement using a standard single-round of replication assay [21]–[23]. HIV-1 viruses were prepared by transfecting HIV-1 proviral clones into 293 cells (producer cells), and infectivity was analyzed by inoculating TZM-bl cells (target cells) with defined amounts of p24 Gag protein of the resultant viruses. We first used the proviral clone NL43 and a Nef-defective mutant (ΔNef) and confirmed that the infectivity of the ΔNef viruses was lower than that of the NL43 wild-type (WT) viruses (Fig. 1B). When added to the producer 293 cells, 2c did not affect the production of WT or ΔNef viruses, even at a high concentration such as 75 µM (Fig. 1C): there was no significant difference in the supernatant p24 Gag protein concentration (upper graph) or the processing of the Gag polyprotein in the cells (lower blots) between the control and 2c-treated cells. However, we found that the infectivity of the WT viruses produced in the presence of 2c was significantly lower than that of the control viruses (Fig. 1D, upper). An inhibitory effect of 2c was detectable at a minimal concentration of 25 µM. Importantly, no such inhibition was observed for the ΔNef viruses, even at a high 2c concentration (75 µM) (Fig. 1D, lower). In the experiment shown in Fig. 1D, WT and ΔNef viruses were inoculated into TZM-bl cells, and the concentration of p24 was adjusted (2 or 4 ng/ml and 8 or 16 ng/ml for WT and ΔNef viruses, respectively) so that the two viruses were similarly infective to the target cells (see Fig. 1B). As the supernatant of proviral plasmid-transfected 293 cells was used as a viral stock, 2c was also present in the culture of target cells (<5 µM). However, 2c did not reduce the infectivity when added to the target cells at a high concentration (10 or 25 µM) together with WT viruses produced in the absence of 2c (Fig. 2A), suggesting that the presence of 2c in the producer cells was essential for its inhibitory effect. Although 2c was added to the producer cells immediately after transfection in Fig. 1D, the inhibitory effect was also observed when 2c was added 24 h after transfection (Fig. 2B). Importantly, 2c did not show any inhibitory effect on the infectivity of Nef^+^ HIV-1 viruses pseudotyped with VSV-G (Fig. 2C), which was consistent with the finding that Nef was dispensable for the infectivity of VSV-G-pseudotyped HIV-1 [24], [25]. Therefore, these results indicated that 2c specifically reduced the infectivity of the wild-type NL43 viruses produced in the presence of Nef.

**Figure 3:**
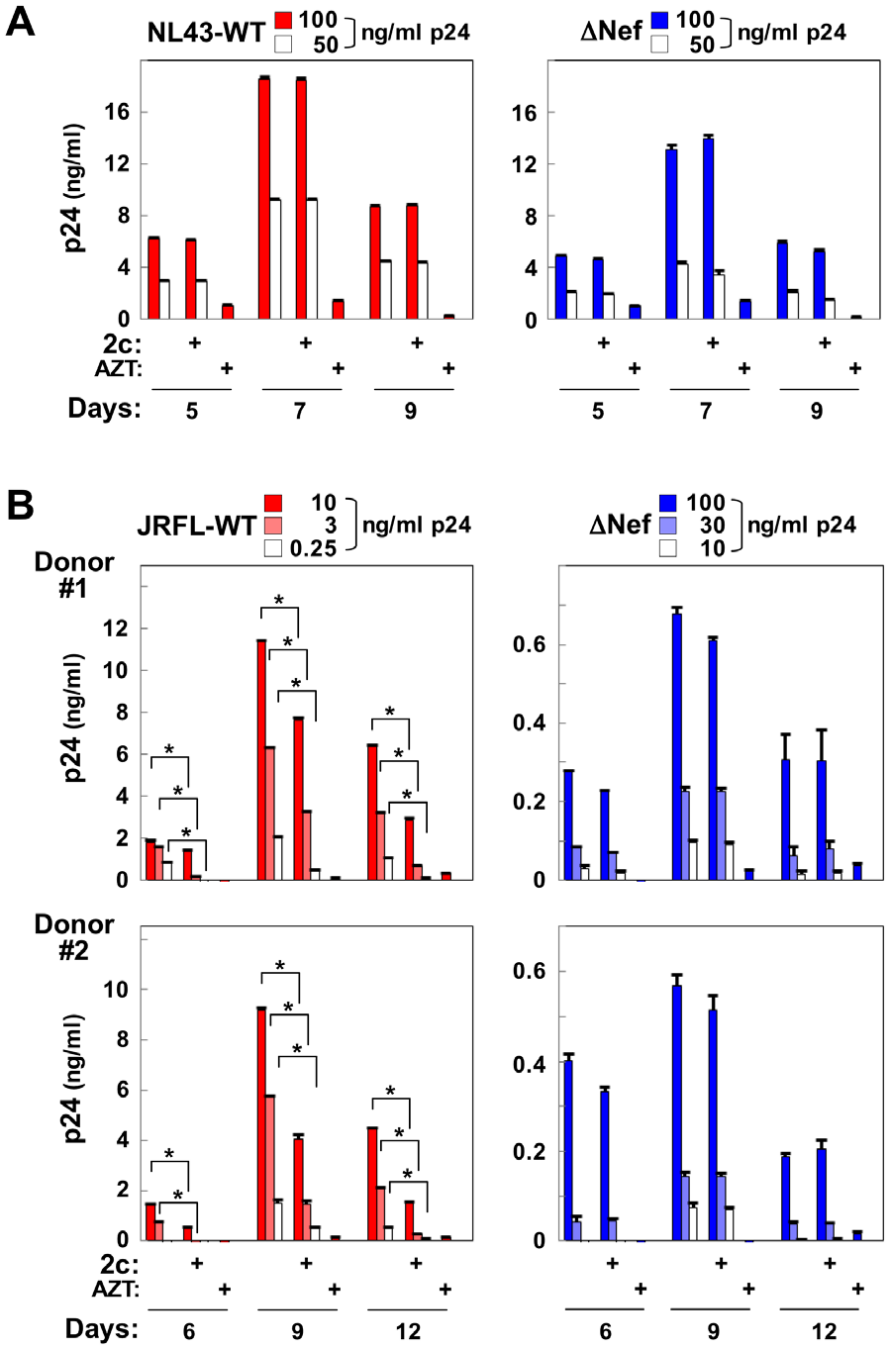
The effect of 2c on the replication of HIV-1. (A) Jurkat cells were infected with either the NL43 wild-type (WT) or Nef-defective (ΔNef) viruses at the indicated concentrations of p24, and cultured in the presence (50 µM) or absence of 2c. AZT was also used at 5 µM. The concentration of p24 in the supernatants (at day 5, 7 or 9) was determined by ELISA. Data are shown as the mean±SD of triplicate assays and are representative of two independent experiments with similar results. (B) Peripheral blood monocyte-derived macrophages were obtained from two different donors, infected with either the JRFL wild-type (WT) or Nef-defective (ΔNef) viruses at the indicated concentrations of p24, and cultured in the presence (50 µM) or absence of 2c. AZT was also used at 5 µM. The concentration of p24 in the supernatants (at day 6, 9 or 12) was determined by ELISA. Data are shown as the mean±SD of triplicate assays. *p<0.05.

We also assessed the effect of 2c on viral replication. 2c decreased by half in the number of viable peripheral blood mononuclear cells after 9 days when used at 50 µM (data not shown). On the other hand, 2c at the same concentration showed no detectable toxicity to 293, TZM-bl, Jurkat T cells and macrophages (data not shown). We therefore used Jurkat and macrophages as target cells. As previously reported [28], the replication of HIV-1 NL43 was independent of Nef in Jurkat T cells (Fig. 3A). Accordingly, 2c failed to inhibit viral replication in the cells (Fig. 3A). However, WT JRFL viruses replicated more efficiently than ΔNef viruses in monocyte-derived macrophages, and 2c significantly reduced the replication of WT viruses (Fig. 3B). The result further supported the idea that the primary target of 2c was Nef.

### The inhibitory effect of 2c requires the proline-rich PxxP motif of Nef

**Figure 4:**
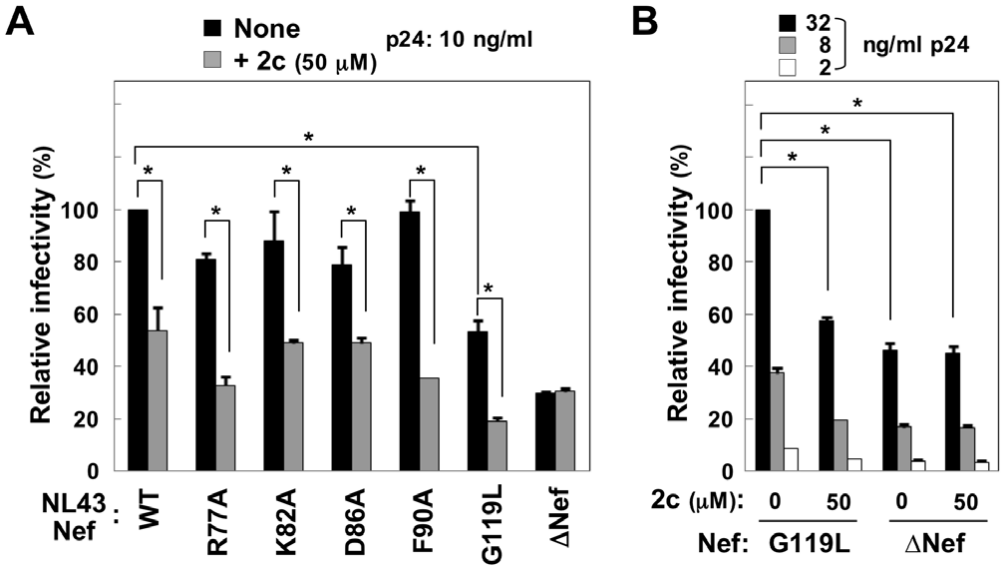
The effect of 2c on the infectivity of NL43 viruses with point amino acid mutations in Nef. (A) The infectivity of the indicated NL43 viruses produced by 293 cells in the absence or presence of 50 µM 2c was determined using TZM-bl cells as the target cells and is expressed as a percentage of the value for the sample on the far left. The amount of p24 inoculated was 10 ng/ml. Wild-type (WT), Nef-defective (ΔNef), or viruses with the indicated amino acid point mutations in Nef (R77A, K82A, D86A, F90A, or G119L) were used. (B) The infectivity of NL43 viruses with the G119L mutation in Nef or ΔNef viruses produced by 293 cells in the absence or presence of 50 µM 2c was determined using TZM-bl cells as the target cells and is expressed as a percentage of the value for the sample on the far left. The amount of p24 inoculated was 2, 8, or 32 ng/ml. (A, B) Data are shown as the mean±SD of triplicate assays and are representative of two independent experiments with similar results. *p<0.05.

Next, we tested the inhibitory activity of 2c on the infectivity of NL43 viruses with point mutations in Nef; i.e., R77A, K82A, D86A, F90A, or G119L [35]. As shown, 2c reduced the infectivity of all these viruses, although to a varying degree (Fig. 4A). Interestingly, the intrinsic infectivity of the NL43-G119L viruses was shown to be low [35] (also see Fig. 4A), but 2c further reduced the infectivity of the mutant viruses to the level of the ΔNef viruses (Fig. 4B). This result supported the conclusion that 2c reduced the infectivity of the NL43 viruses in a Nef-dependent manner.

**Figure 5:**
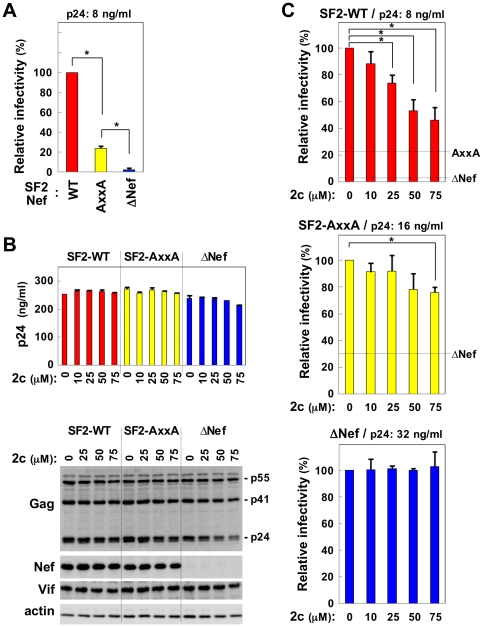
The effect of 2c on the infectivity of SF2 wild-type, Nef-defective, and Nef PxxP motif-disrupted viruses. (A) The infectivity of the SF2 wild-type (WT), Nef-defective (ΔNef), and Nef PxxP motif-disrupted viruses (AxxA) was compared by inoculating them into the target TZM-bl cells at a concentration of 8 ng/ml p24 and is expressed as a percentage of the value for the sample on the far left. Data are shown as the mean±SD of triplicate assays and are representative of two independent experiments with similar results. *p<0.05. (B) 2c was added to 293 cells producing SF2-WT, ΔNef, or AxxA viruses at the indicated concentrations for 2 days, and the concentration of p24 Gag protein in the supernatants was determined by ELISA (bar graph). Data are shown as the mean±SD of triplicate assays and are representative of two independent experiments with similar results. Alternatively, the producer cells were lysed and analyzed for the expression of Gag, Nef, and Vif by Western blotting (lower blots). The actin blot was used as a loading control. (C) The infectivity of SF2-WT (top), AxxA (middle), or ΔNef viruses (bottom) produced by 293 cells in the absence or presence of the indicated concentrations of 2c was determined using TZM-bl cells as the target cells. The WT, AxxA, and ΔNef viruses were inoculated by changing the concentration of p24 (8 ng/ml, 16 ng/ml, and 32 ng/ml for WT, AxxA and ΔNef viruses, respectively) so that these viruses were similarly infective to the target cells. Infectivity is expressed as a percentage of the value for the sample on the far left. In the top panel, the infectivity values of the AxxA and ΔNef viruses produced at the same concentration of p24 (i.e., 8 ng/ml) are also shown as a reference. In the middle panel, the infectivity values of the ΔNef viruses produced at the same concentration of p24 (i.e., 16 ng/ml) are also shown. Data are shown as the mean±SD of triplicate assays and are representative of three independent experiments with similar results. *p<0.05.

The dileucine motif of Nef (^164^LL^165^) that is required for CD4 downregulation is also required for the enhancement of infectivity [3], [36]. However, it was unlikely that the inhibitory activity of 2c was mediated through the motif, as 2c did not inhibit CD4 downregulation [31]. On the other hand, Nef has another characteristic motif; i.e., a proline-rich PxxP motif, and the substitution of the proline residues for alanine residues (AxxA) is known to result in reduced viral infectivity [3]. Thus, we tested whether 2c further reduced the infectivity of Nef-AxxA viruses as it did with G119L mutant viruses (see Fig. 4B). To test whether 2c is also effective against Nef derived from an additional HIV-1 strain, we used an HIV-1 JRFL construct in which *nef* gene was replaced with that of the SF2 strain Nef or its AxxA mutant [30] in the subsequent experiments. First, as expected, the infectivity of the Nef- AxxA viruses was lower than that of the wild-type (WT) viruses, although it was still higher than that of the ΔNef viruses (Fig. 5A). As was the case with the NL43 viruses (see Fig. 1C), 2c did not affect viral production in the JRFL-SF2 Nef viruses (Fig. 5B): there was no change in the amount of p24 Gag protein in the supernatants (upper graph), the processing of the Gag polyprotein, or the expression of Nef or another viral protein, Vif, (lower blots) between the control and 2c-treated cells. Moreover, as was the case with the NL43 viruses (see Fig. 1D), 2c significantly reduced the infectivity of the produced JRFL-SF2 Nef WT viruses, but not that of the ΔNef viruses (Fig. 5C). However, we found that 2c minimally affected the infectivity of the Nef-AxxA mutant viruses (Fig. 5C, middle), which was in contrast with the finding that it further reduced the infectivity of the Nef-G119L mutant viruses (see Fig. 4B). These results suggested that the inhibitory activity of 2c is mediated, at least in part, through the proline-rich motif of Nef.

### 2c binds directly to Nef in a similar manner to the Hck SH3 domain

**Figure 6:**
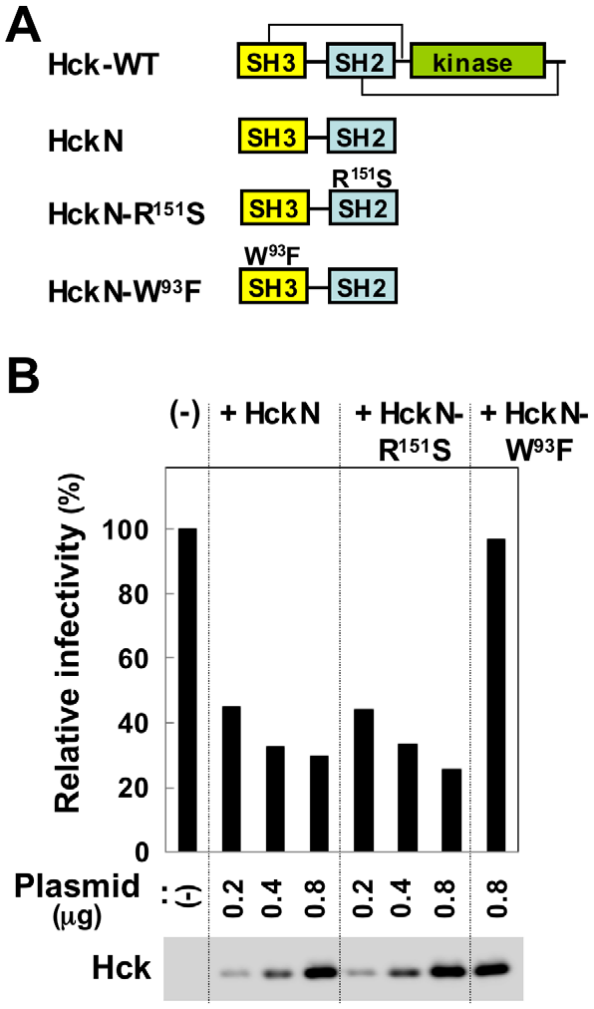
The effects of the overexpression of mutant forms of Hck on viral infectivity. (A) The mutant forms of Hck used are shown schematically. HckN lacks the kinase domain and the two intra-molecular interactions present in the wild-type (WT) Hck. HckN-based HckN-R151S and HckN-W93F have amino acid substitutions in their SH2 and SH3 domain, respectively. (B) The 293 cells were transfected with the NL43 wild-type proviral plasmid or co-transected with the indicated amount of plasmid (HckN, HckN-R151S, or HckN-W93F). The infectivity of the viruses produced in the supernatants was determined using TZM-bl cells as the target cells and is expressed as a percentage of the value for the sample on the far left (bar graph). The amount of p24 inoculated was 8 ng/ml. Alternatively, the producer 293 cells were lysed and analyzed for the expression of the mutant Hck proteins by Western blotting (blot).

Although 2c was the first small molecule to be found to reduce the Nef-mediated infectivity of HIV-1, the overexpression of mutant forms of Hck in viral producer cells was also reported to result in reduced viral infectivity [37]. Hck is a cellular tyrosine kinase, and its SH3 domain has been shown to bind to Nef with high affinity [14]–[16], although its pathological significance is not yet understood. It is also known that the SH3 domain forms an intra-molecular interaction with the linker region of Hck [15], [16] (also see Fig. 6A). Thus, the SH3 domain of mutant Hck, which lacks the linker region and the subsequent kinase domain (see Fig. 6A, HckN), is devoid of the intra-molecular interaction, and is thought to more efficiently bind to Nef and thereby reduce viral infectivity. Indeed, when co-expressed with the NL43 proviral clone, HckN and HckN-R151S, which carries a mutation in its SH2 domain, but not HckN-W93F, which carries a mutation in its SH3 domain, significantly reduced the infectivity of viruses produced from the cells (Fig. 6B).

**Figure 7:**
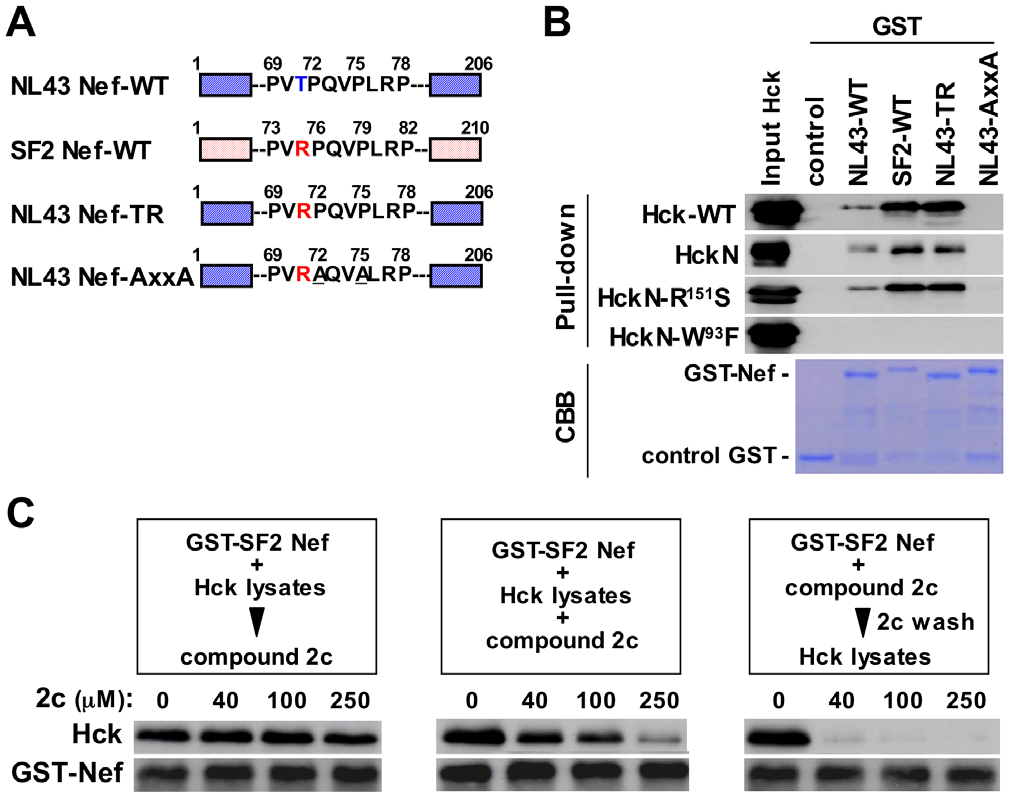
The effect of 2c on binding between Nef and Hck. (A) The Nef proteins fused to GST are shown schematically. In addition to the wild-type (WT) SF2 and NL43 strain Nef, the NL43-TR mutant, which contained a T71R amino acid substitution, and another NL43 AxxA mutant, in which the PxxP motif was disrupted (P72A and P75A substitutions), were used. (B) The resins to which the control GST or indicated GST-Nef fusion proteins were bound were incubated with the lysates of 293 cells expressing the indicated Hck protein. The amount of Hck bound to the resins was determined by Western blotting (pull-down assay). To confirm the equal expression of these Hck proteins in the 293 cells, equal amounts of each cell lysate were analyzed (Input Hck). Moreover, the amounts of the GST and GST-Nef fusion proteins bound to the resins were verified by the elution from the resins followed by SDS-PAGE/Coomassie brilliant blue (CBB) staining. (C) Three different competitive pull-down assays were performed. In the experiment shown in the left panel, the resins to which the GST-SF2 Nef fusion proteins were bound were incubated with the lysates obtained from the 293 cells expressing the wild-type Hck for 3 h, and then 2c was added to the mixture at the indicated concentration. In the experiment shown in the middle panel, the resins to which the GST-SF2 Nef fusion proteins were bound were incubated with the lysates of 293 cells expressing the wild-type Hck and the indicated concentration of 2c. In the experiment shown in the right panel, the resins to which the GST-SF2 Nef fusion proteins were bound were first incubated with the indicated concentration of 2c for 4 h and then washed to remove unbound 2c. Then, the resins were incubated with the lysates of 293 cells expressing the wild-type Hck. The amount of Hck bound to the resins was determined by Western blotting (upper blots). The GST-Nef blot was used as a loading control (lower blots). Data shown are representative of two independent experiments with similar results.

Based on these results, we hypothesized that 2c inhibits viral infectivity in a similar manner to mutant Hck. To this end, we examined whether 2c and Hck compete to bind to Nef using an *in vitro* pull-down assay. First, we performed a pull-down assay with various combinations of GST-Nef fusion proteins (Fig. 7A) and the Hck proteins described above. As a result, we found that the wild-type (WT) NL43 Nef bound to the wild-type (WT) Hck, HckN, and HckN-R151S, but not Hck-W93F, which had a mutation in its Nef-binding SH3 domain (Fig. 7B). In contrast, the PxxP motif-disrupted AxxA mutant did not bind to any of these Hck proteins (Fig. 7B), confirming that the pull-down system specifically detected Nef-Hck binding. As the affinity of the SF2 strain Nef for Hck was higher than that of NL43 strain Nef, which was due to the different amino acid present within the PxxP motif (Figs. 7A and B, NL43 Nef-TR mutant with a T71R substitution), we used SF2 Nef in the following experiments. Among three different competitive pull-down assays, the pre-incubation of Nef with 2c most effectively inhibited the binding of Hck to Nef (Fig. 7C, right). We therefore concluded that both the Hck SH3 domain and 2c directly bind to Nef and that their binding sites overlap.

**Figure 8:**
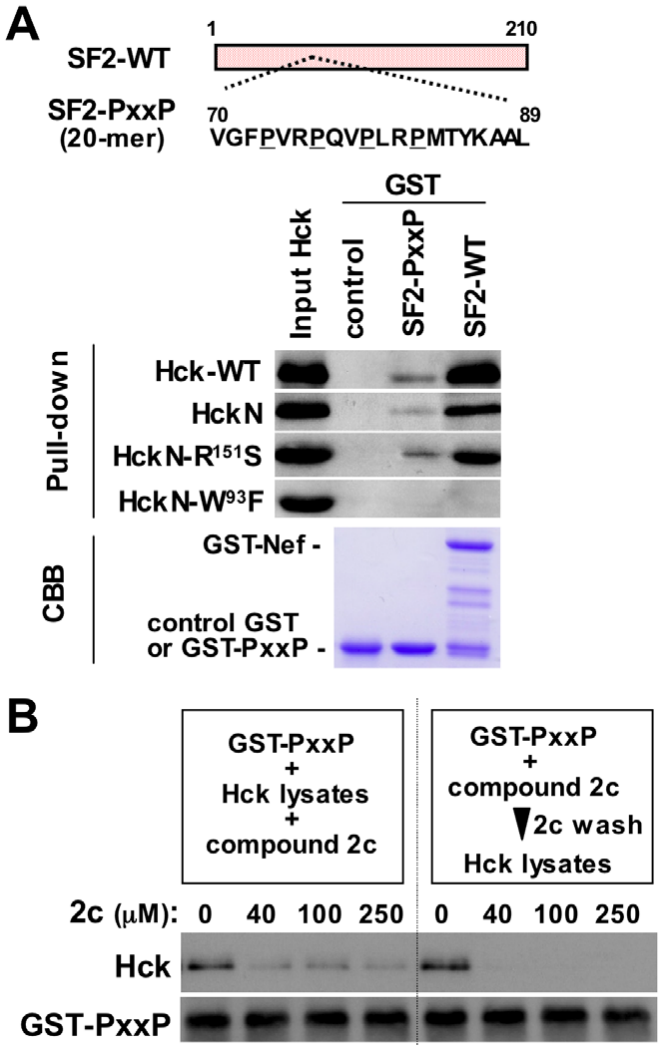
The effect of 2c on the binding between Nef PxxP motif-containing peptides and Hck. (A) The Nef peptide fused to GST is shown schematically. The 20 amino acid peptide derived from the PxxP motif of SF2 Nef was used (the proline residues are underlined). The resins to which the control GST, GST-SF2 Nef-PxxP peptides (SF2-PxxP), or GST-intact SF2 Nef (SF2-WT) fusion proteins were bound were incubated with the lysates of 293 cells expressing the indicated Hck protein. The amount of Hck bound to each resin was determined by Western blotting (Pull-down). To verify the equal expression of these Hck proteins in the 293 cells, equal amounts of each cell lysate were analyzed (Input Hck). Moreover, the amounts of the GST and GST-Nef fusion proteins bound to the resins were verified by eluting from the resins followed by SDS-PAGE/Coomassie brilliant blue (CBB) staining. (B) Two different competitive pull-down assays were performed. In the experiment shown in the left panel, the resins to which the GST-SF2 Nef-PxxP peptides were bound were incubated with the lysates of 293 cells expressing the wild-type Hck and the indicated concentration of 2c. In the experiment shown in the right panel, the resins to which the GST-SF2 Nef-PxxP peptides were bound were incubated with the indicated concentrations of 2c for 4 h and then washed to remove unbound 2c. Then, the resins were incubated with the lysates of 293 cells expressing the wild-type Hck. The amount of Hck bound to the resins was determined by Western blotting (upper blots). The GST-Nef blot was used as a loading control (lower blots). Data shown are representative of two independent experiments with similar results.

To further confirm the above-mentioned conclusion, we used a GST fusion protein containing a 20-mer peptide derived from the PxxP motif of SF2 Nef (Fig. 8A, SF2-PxxP). As shown, the observed binding of the SF2-PxxP peptides to Hck was specific, albeit weak, in comparison with that of the full-length Nef, since it was detected with the wild-type Hck, HckN, and HckN-R151S, but not with the Nef binding-deficient HckN-W93F (Fig. 8A). Importantly, 2c inhibited the binding of Hck to the Nef-PxxP peptide, and its inhibitory effect was more marked when the Nef-PxxP peptide was pre-incubated with 2c prior to its incubation with Hck (Fig. 8B). This result suggests that 2c binds to Nef, at least in part, through the region including the PxxP motif, which is consistent with the finding that unlike the wild-type viruses, the infectivity of the PxxP motif-disrupted AxxA mutant viruses was minimally affected by 2c (see Fig. 5C).

**Figure 9:**
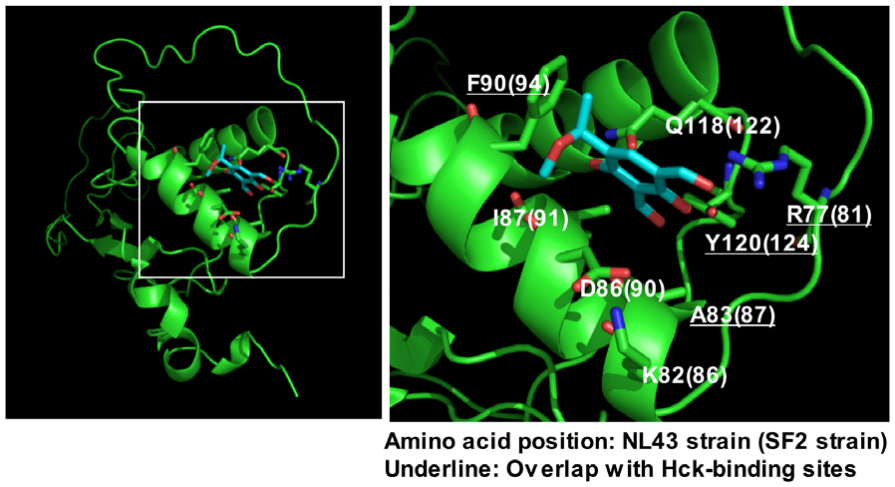
The 2c-Nef docking model. The amino acids that are predicted to be involved in the interaction between Nef and 2c are indicated. The positions of these amino acids in the NL43 strain and SF2 strain are shown. The amino acids predicted to interact with the Hck SH3 domain are underlined [38].

Finally, a computer-assisted simulation of the 2c-Nef docking model supported the idea that 2c binds directly to Nef and suggested that R77, K82, A83, D86, I87, F90, Q118, and Y120 (positions are based on the sequence of NL43 strain Nef) may be responsible for this binding (Fig. 9). Among them, R77 lies within the PxxP motif (–PVTPQVPLR^77^ P–, the proline residues are underlined). On the other hand, molecular modeling also identified several residues in Nef that are responsible for its binding to Hck, such as P72, P75, R77, A83, F90, W113, His116, and Y120 [38]. Among them, R77, A83, F90, and Y120 were also found in the 2c-Nef docking model (Fig. 9, underlined), supporting the finding that 2c inhibits the binding of Hck to Nef or Nef PxxP motif-derived peptides (Figs. 7C and 8B). In summary, the present study revealed that the compound 2c reduced the infectivity of HIV-1 viruses and suggested that its inhibitory activity is mediated by its direct binding to Nef.

**Figure 10:**
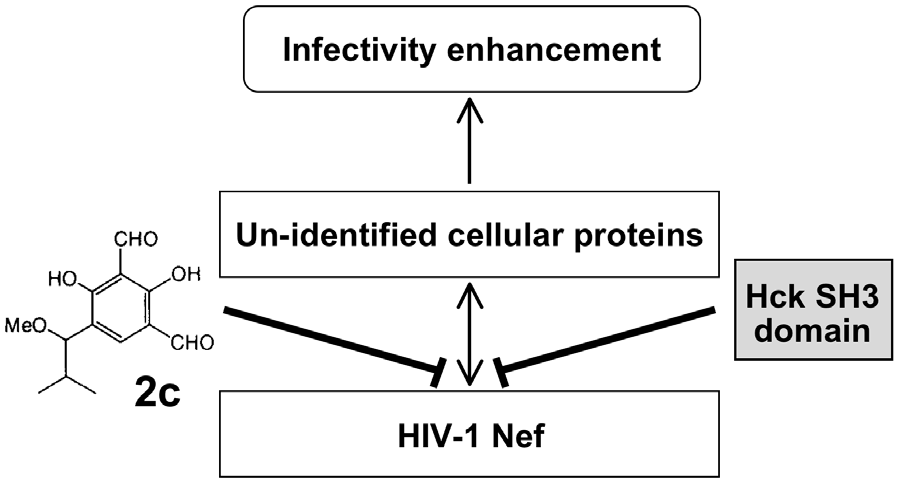
A model of the inhibitory effect of 2c. Both 2c and the Hck SH3 domain bind directly to Nef and reduce viral infectivity, probably by inhibiting the interaction of Nef with an unidentified cellular protein(s).

It remains to be determined exactly how 2c reduces Nef-mediated infectivity enhancement. Given that both 2c and the Hck SH3 domain bind directly to overlapping domains of Nef and reduce viral infectivity, we speculate that their inhibitory effects are due to the inhibition of the interaction of Nef with host proteins (Fig. 10). One of the candidates for such a host protein is p21-activated kinase 2, PAK2, the association of which depends on the Nef PxxP motif [39]. However, we did not observe any inhibitory effect of 2c on the association of Nef with PAK2 activity or its downstream effector functions (data not shown), and the Nef-PAK2 association is dispensable for the enhancement of infectivity by the viral protein [40]. Another candidate is the GTPase dynamin 2 whose interaction with Nef was implicated in enhancing viral infectivity [41]. However, again, we did not observe any significant inhibitory effect of 2c on the binding of Nef to dynamin 2, which was assessed using a co-immunoprecipitation assay (data not shown). Thus, the inhibitory activity of 2c observed in this study appears to be independent of these host proteins. The inhibitory compound 2c is a useful chemical probe for investigating the underlying molecular mechanism by which Nef enhances the infectivity of HIV-1, and in particular, for identifying the host proteins involved in the process.

Recently, a single-domain antibody (sdAb) that binds to Nef was reported [42]. Although the binding domains in Nef remained unclear, anti-Nef sdAb was also shown to reduce *in vitro* viral infectivity [42]. Therefore, to clarify whether viral infectivity enhancement by Nef accounts for the high *in vivo* viral load observed in the presence of Nef, it is necessary to test the effects of 2c, a more potent analog, and/or the anti-Nef sdAb in animal models such as HIV-1-infected humanized mice.

## Materials and Methods

### The compound 2c preparation

Some of the 2c was prepared by Kyowa Hakko Kogyo (Tokyo, Japan), as described previously [43], whilst the rest (a large quantity) was prepared by Sai Advantium Pharma (Hyderabad, India). Both preparations were dissolved in DMSO and had an equivalent inhibitory effect on HIV-1 infectivity (data not shown).

### Proviral plasmids

The provial NL43 plasmid and its derivatives, which had mutations in the Nef gene (ΔNef, R77A, K82A, D86A, F90A, and G119L), were prepared as described previously [35]. The Env-defective mutant (pNL-Kp) and VSV-G expression plasmid were prepared as described previously [44]. The proviral JRFL plasmid was provided by Y. Koyanagi (Kyoto University, Kyoto, Japan) [45]. We also prepared the proviral JRFL plasmid, in which the Nef gene was disrupted (ΔNef) or replaced with the PxxP motif-disrupted AxxA mutant [30].

### Hck plasmids

The p56Hck cloned into the pcDNA3.1 vector (Invitrogen) was prepared as described previously [18]. The mutant forms of Hck cloned into the pCAGGS vector (HckN, Hck-R151S, and Hck-W93F; see Fig. 6A) were provided by M. Matsuda (Kyoto University, Kyoto, Japan) [37].

### GST fusion plasmids

The control GST and GST-Nef fusion plasmids (the wild-type NL43, NL43 Nef-TR mutant, NL43 Nef-AxxA, and the wild-type SF2; see Fig. 7) were prepared as described previously [30]. We also prepared a GST-SF2 Nef-PxxP plasmid, which expressed a 20-mer peptide derived from the PxxP motif of SF2 Nef (see Fig. 8). The cDNA containing the motif was amplified by PCR using the following primers (5′-GGATCCGTGGGTTTTCCAGT-3′ and 5′-GTCGACCTATAAAGCTGCCT-3′), cloned into the pCR2.1 vector (Invitrogen), sequenced using the BigDye Terminator v3.1 Cycle Sequencing kit (Applied Biosystems) and the ABI PRISM 3100 Genetic Analyzer (Applied Biosystems), and cloned into the pGEX-6P-1 bacterial expression vector (GE Healthcare).

### Virus preparation

HEK293 cells (Invitrogen) were maintained in DME medium supplemented with 10% FCS and used as viral producer cells. The 293 cells were seeded onto 12-well tissue culture plates at a density of 1.8×10^5^ cells/well and transfected with 1.6 µg/well of various proviral HIV-1 plasmids using 4 µl/well Lipofectamine 2000 reagent (Invitrogen). To prepare VSV-G-pseudotyped viruses (see Fig. 2C), cells were transfected with 0.5 µg/well Env-defective mutant plasmid (pNL-Kp) and 1.0 µg/well VSV-G expression plasmid. In a selected experiment (see Fig. 6B), the cells were co-transfected with 0.8 µg/well pNL43 plasmid and 0.2, 0.4, or 0.8 µg/well of one of the mutant forms of Hck (HckN, HckN-R151S, or HckN-W93F). The total amount (1.6 µg/well) of the plasmid was normalized using the pCAGGS empty vector. After 6 h of transfection, the culture medium was replaced with fresh medium, and the cells were cultured for an additional 48 h in the presence or absence of 2c at the indicated concentrations. In a selected experiment (see Fig. 2B), 2c was added to the culture 24 h after transfection. Then, the supernatants containing the viruses were clarified by brief centrifugation, and viral production was assessed by measuring the concentration of p24 Gag protein in the supernatants using the RETROtek p24 Antigen ELISA kit (ZeptoMetrix). Viral production was also assessed by analyzing the expression of viral proteins in the cells by Western blotting. The preparation of the total cell lysates and Western blotting were performed essentially as described previously [17], [18], [30]. Briefly, the cells were lysed on ice with Nonidet P-40 lysis buffer (1% Nonidet P-40, 50 mM Tris, and 150 mM NaCl) containing protease inhibitors (1 mM EDTA, 1 µM PMSF, 1 µg/ml aprotinin, 1 µg/ml leupeptin, and 1 µg/ml pepstatin). Total cell lysates were then subjected to Western blotting. The antibodies used were as follows: anti-Gag (#65-004; BioAcademia, Osaka, Japan), anti-Nef (#2949; NIH AIDS Research & Reference Program), anti-Vif (#319; NIH AIDS Research & Reference Program), and anti-actin (#C-2; Santa Cruz). The detection was performed with HRP-labeled secondary antibodies (GE Healthcare), the Immunostar LD Western blotting detection reagent (Wako, Osaka, Japan), and an image analyzer (ImageQuant LAS 4000; GE Healthcare).

### Infectivity assay

TZM-bl cells (NIH AIDS Research & Reference Program) were maintained in DME medium supplemented with 10% FCS and used as viral target cells. TZM-bl cells were seeded onto 96-well tissue culture plates at a density of 6×10^3^ cells/well and challenged with serially diluted viruses normalized for the concentration of p24 Gag protein. The supernatant of the proviral plasmid-transfected 293 cells was used as a viral stock and diluted with DME medium containing 10% FCS and 20 µg/ml DEAE-dextran (MP Biomedicals, Solon, OH). The diluted viruses were then added to the target cells (150 µl/well) overnight, and the culture medium was replaced with fresh DME medium containing 10% FCS and incubated for 48 h. In a selected experiment (see Fig. 2A), 2c was added to TZM-bl cells together with the diluted viruses. Viral infectivity was assessed by measuring the HIV-1 Tat-mediated induction of β-galactosidase activity in the target cells using a β-Galactosidase Enzyme Assay System (Promega). The absorbance of the wells was measured at 420 nm using a Multiskan microplate reader (Thermo Electron).

### Replication assay

The replication assay with macrophages was performed essentially as described previously [46]. Heparinized venous blood was collected from healthy donors, after informed consent was obtained in accordance with the Declaration of Helsinki. The approval for this study was obtained from the Kumamoto University Medical Ethical Committee. Mononuclear cells obtained using LSM reagent (MP Biomedicals) were suspended into RPMI1640 medium-1% FCS at 1×10^6^ cells/ml and seeded into 24-well plates. Monocytes were enriched by adherence to plates for 1 h at 37°C, and non-adherent cells were removed by extensive washing with PBS. Then, the adherent monocytes were differentiated into macrophages by culturing with RPMI1640-10% FCS containing 100 ng/ml rhM-CSF (a gift from Morinaga Milk Industry, Kanagawa, Japan). After 3 days, the cultures were replaced with fresh complete media and incubated for another 3 days. The purity of the day 6-macrophages prepared by this method was routinely more than 95% when assessed by the expression of CD14 (data not shown). Then, macrophages were incubated with 250 µl of the 293 cell supernatants containing JRFL HIV-1 viruses for 2 h at 37°C. Either 2c or DMSO was added to the incubation together with the diluted viruses. AZT (NIH AIDS Research & Reference Program) was used as a positive control. The cells were washed twice with PBS to remove unbound viruses and cultured with RPMI1640-10% FCS containing rhM-CSF in the presence or absence of 2c or AZT. One-half of the culture media was replaced with the complete media every 3 days. The culture supernatants collected at day 6, 9 and 12 were analyzed for the concentration of p24 Gag proteins by ELISA to monitor viral replication.

Jurkat cells were also used in this study. The cell pellet (1×10^6^ cells) were incubated with 500 µl of the 293 cell supernatants containing NL43 viruses for 2 h at 37°C. Either 2c or DMSO was added to the incubation together with the diluted viruses. AZT was used as a positive control. The cells were washed twice with PBS, resuspended into 1 ml of RPMI1640-10% FCS, and cultured for 3 days in the presence or absence of 2c or AZT. Then, the culture were diluted (1/5) with RPMI1640-10% FCS, and cultured for another 2 days in the presence or absence of 2c or AZT. The concentration of p24 in the culture supernatants of day 5, 7 and 9 was analyzed as above.

### GST pull-down assay

The control GST and GST-Nef fusion proteins cloned in the pGEX-6P-1 vector were expressed in *E. coli* BL21 cells (GE Healthcare). The cells were grown in LB medium containing 50 µg/ml ampicillin, before being induced with 1 µM IPTG (Sigma). The expression-induced cells were harvested and lysed with BugBuster Protein Extraction Reagent containing 1 U/ml rLysozyme and 25 U/ml Benzonase Nuclease (all from Novagen). The cleared lysates were then incubated with GST-Bind Resin (Novagen). After extensive washing with GST Bind/Wash Buffer (Novagen), the resin was incubated with the total cell lysate of the 293 cells transfected with the expression plasmid for Hck for 12 h. In the competitive pull-down assay, we employed the following 3 protocols: (1) the concurrent addition of 2c and Hck-containing lysates to the GST-Nef-bound resin, (2) the addition of Hck-containing lysates for 3 h followed by the addition of 2c, (3) the addition of 2c at the indicated concentrations for 4 h followed by the addition of the Hck-containing lysates. The incubation of the above mixtures was carried out at 4°C in Nonidet P-40 lysis buffer (1% Nonidet P-40, 50 mM Tris, and 150 mM NaCl) containing protease inhibitors (1 mM EDTA, 1 µM PMSF, 1 µg/ml aprotinin, 1 µg/ml leupeptin, and 1 µg/ml pepstatin). After extensive re-washing with complete Nonidet P-40 lysis buffer, the resin was boiled with SDS-PAGE sample buffer, and the eluates were analyzed for the presence of Hck by Western blotting with anti-Hck antibodies (clone 18; Transduction Laboratories).

### The 2c-Nef docking model

We predicted the complex structures of Nef and 2c by homology modeling and docking simulation using the Molecular Operating Environment (MOE) ver. 2007.09. (Chemical Computing Group, Canada). First, homology modeling [47]–[49] was used to construct the model structure of HIV-1 Nef SF2 strain using its NMR structure (PDB code: 2NEF) [50] as a template. During the modeling, energy calculations were performed with the AMBER ff99 force field [51] and the GB/VI implicit solvent energy function [52]. Next, docking simulation of 2c with the homology model of Nef was achieved with the ASEDock module [53]. The initial structure of 2c was generated with the Molecular Builder module. Then, we searched for the binding site of 2c with the Site-Finder module. During the simulation, the energy calculations were performed with the MMFF94x force field [54], [55] and the GB/VI implicit solvent energy function [52]. During the docking simulation, movement of the main chain atoms around 4.5 Å of the ligand binding site in Nef was restrained with a harmonic potential of 100 kcal/mol/Å^2^, while the atoms in compound 2c were not constrained. In this study, the structure with the lowest score was selected for the model.

### Statistical analysis

The statistical significance of differences between assay groups was determined using Mann-Whitney *U* test. *p* values less than 0.05 were considered significant.
